# Unidirectional Electron-Tunnelling Flexible PDMS Strain Sensor with Aligned Carbon Nanotubes

**DOI:** 10.3390/s23208606

**Published:** 2023-10-20

**Authors:** Tim Mike de Rijk, Sascha Schewzow, Andreas Schander, Walter Lang

**Affiliations:** Institute for Microsensors, Actuators and Systems, University Bremen, 28359 Bremen, Germany; s_g2wf3e@uni-bremen.de (S.S.); aschander@imsas.uni-bremen.de (A.S.); wlang@imsas.uni-bremen.de (W.L.)

**Keywords:** aligned carbon nanotubes, electron tunnelling, flexible sensors, nanomaterials, unidirectional sensing

## Abstract

High-aspect-ratio carbon nanotubes can be directly mixed into polymers to create piezoresistive polymers. Reducing the cross-sensitivity and creating unidirectional sensitive sensors can be achieved by aligning the nanotubes before they are cured in the polymer layer. This research presents and characterises this alignment of carbon nanotubes inside polydimethylsiloxane and gives the corresponding strain sensor results. The influence on the alignment method, as well as the field strength, frequency and time is shown. An analytical model is created to investigate the sensor’s behaviour and determine the effect of electron-tunnelling in the sensor. A numerical model gives insight into the necessary applied field strength, frequency and time to facilitate alignment in viscous liquids. The experimental data show a two-phase piezoresistive response; first, a linear strain response, after which the more dominant electron-tunnelling piezoresistive phase starts with high gauge factors up to k ≈ 4500 in the preferential direction, depending on the carbon nanotube concentration. Gauge factors in the orthogonal direction remain low (k ≈ 22). Finally, the dynamic stability of the sensors is proven by exposing the sensors to a cyclic strain. Small initial drifts are observed but appear to stabilise after several cycles.

## 1. Introduction

Carbon nanotubes (CNTs) have emerged as a versatile and promising material for enhancing the properties of polymers in various applications, particularly in the field of piezoresistive sensors. The integration of high-aspect-ratio CNTs into polymer matrices has started a whole new field of piezoresistive polymers capable of accurately sensing mechanical deformations and strains. These sensors are applied in a very wide range of fields, including aerospace [1,2,3], structural health monitoring [4,5,6,7], medical devices [8,9] and wearable technology [10,11,12,13,14]. However, one of the challenges in harnessing the full potential of CNT-based piezoresistive sensors lies in optimising the alignment of CNTs within the polymer matrix.

The orientation of the CNTs within their host material can be altered by applying an external electric field. The possible advantages of aligned CNTs can already be intuitively visualised to create connections of high-aspect-ratio CNTs over long, predefined directions. Thermal or electrical conduction paths can be created in a preferred direction, where in all other directions, the conductivity is decreased. These selective conductive paths can be utilised for directional strain sensing with very high gauge factors. Regular carbon nanotube-based sensors often feature relatively low gauge factors (in the order of 0.5–4) [14,15,16,17]. Aligned carbon nanotube polymer layers show up to 10-fold resistance differences in the orthogonal direction, proving a preferential direction inside the polymer [18,19]. Measured gauge factors of aligned carbon nanotube compounds show a far greater potential than those of regular other metallic piezoresistive materials [20,21].

Even higher sensitivities for very small strain regions (up to 0.10%) are found in the literature that are based on piezotronic tunnelling junctions and shown to have a gauge factor up to $4.8\;\times \;10^{5}$ [20].

This study aims to investigate, characterise and optimise the alignment of carbon nanotubes within polydimethylsiloxane and assesses its effects on the performance of piezoresistive sensors. This alignment of carbon nanotubes in a specified direction minimises the cross-sensitivity of the sensor. An ideal sensor features no electrical connections in all other directions and only reacts to the direction of the applied strain. The experimental data are supported by extensive numerical and analytical models, yielding key insights into the working principle of the carbon nanotube alignment due to an applied electric field and the possible high sensitivities due to formed electron-tunnelling connections.

## 2. Materials and Methods

Polydimethylsiloxane (PDMS) Slygard 184 was chosen as a suitable polymer to form the basis of the piezoresistive sensing layer. The main reason for this choice was the fact that the elastomer has no additional solvent that needs to evaporate whilst curing the final layer. It was expected that this removal of solvent would have negatively influenced the alignment quality. Additionally, the molecular structures of elastomers (like PDMS) are comprised of very loose, wide and long polymer chains, causing their flexibility properties. PDMS was chosen as the ideal polymer as no additional solvents were needed and it is well known in the literature and IC technology industry.

The sensor fabrication was completely performed inside a clean room with a regulated temperature and humidity. The characterisation of the sensors was performed in a climate-controlled laboratory. It is known that humidity changes the resistivity of carbon nanotube–polymer layers [22,23]. To fabricate and test all samples inside climate-controlled environments ensured this effect was minimised.

**Figure 1:**
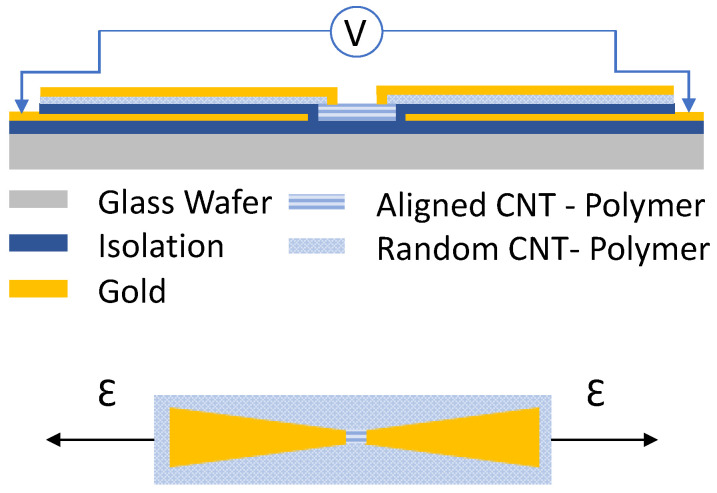
View of the individual sensor layers. The top part shows the fabrication layers on top of the glass wafer. The alignment electrodes are isolated with a polyimide layer (purple). The measurement electrodes are sputtered on top of the cured CNT-PDMS layer. After the sensors are complete, they are detached from the wafer, leaving the glass wafer, alignment electrodes and polyimide layer behind. The bottom part shows a top-view of a single strain sensor with aligned CNTs only between the two electrodes while the rest of the sensor remains randomly dispersed.

The manufacturing process needed two sets of electrodes: alignment electrodes and measurement electrodes. The first were sputtered directly onto a 4-inch glass wafer and isolated by a 5 μm polyimide layer. This isolation was necessary to ensure no connections were possible between the alignment electrodes via the nanotube–polymer mixture, which could cause short circuits and stop the alignment process. The CNT-PDMS mixture was spin-coated directly (without any surface treatment) on top of the polyimide layer. The carbon nanotubes (Sigma-Aldrich Chemie GmbH, Taufkirchen Germany) in this research featured an outside diameter (OD) of 50–90 nm with an aspect ratio >100. The CNTs were mixed with the direct mixing approach, which was shown in previous work [24]. The gold contact pads of the alignment electrodes were freed of polymer and a voltage was applied to the uncured PDMS layer to align the CNTs. Afterwards, the piezoresistive layer was cured at 90 ${}^{\circ }$C for 45 min. A low curing temperature was chosen to minimise the induced stress within the polymer layer. The second set of electrodes were the interdigital electrodes used for measuring the sensor response. This chrome–gold layer was deposited via the sputtering process and structured with standard lithography processes. The only exception was that the photoresist was pre-baked in an oven to slowly ramp the temperature to 90 ${}^{\circ }$C. The standard process would allow the resist to cure on a hotplate, but previous experience showed the layers underneath to crack due to the high temperature expansion coefficient of the PDMS. After the top interdigital electrodes were structured, the sensors were cut and easily removed from the bottom polyimide layer. A simplified version of the sensor layers is shown in Figure 1.

**Figure 2:**
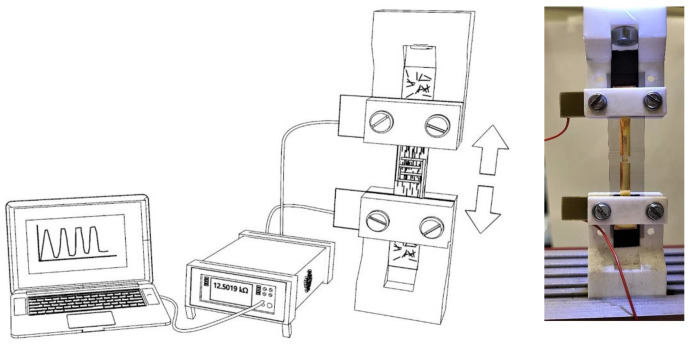
Measurement setup for characterising the strain sensors. The samples are placed inside a custom-made gripper (shown in previous work [24]) and connected to the digital multimeter for continuous resistance measurement. The right-side figure shows an actual sample.

The sensor was based on the fact that when applying a tensile strain, the distance between the conductive particles increases and the conductivity decreases. Ideally, with the aligned CNTs, the sensitivity is increased in the preferred direction and reduced to zero in all others. The individual sensors were placed in a custom-made gripper with copper plates to continuously measure their resistance (shown previously in [24]). The whole setup was placed in the CONDOR 100 Bond tester (XYZTEC), which is completely programmable to preciously pull the samples apart while measuring the applied force. The speed was 20 μm/s and the samples were stretched with up to a 1.0% strain. The resistance was continuously measured with a digital multimeter (Keithley DMM6500). An overview of the measurement setup and a picture of the actual setup with a strain sensor is shown in Figure 2.

The experimental data were supported by both numerical and analytical models. The first model was focused on the principle of rotating individual carbon nanotubes in a liquid medium and the associated electric field strengths, frequencies and viscosity of the liquid. The analytical model detailed the possible sensor piezoresistive responses due to electron tunnelling and loss of conductive networks.

**Figure 3:**
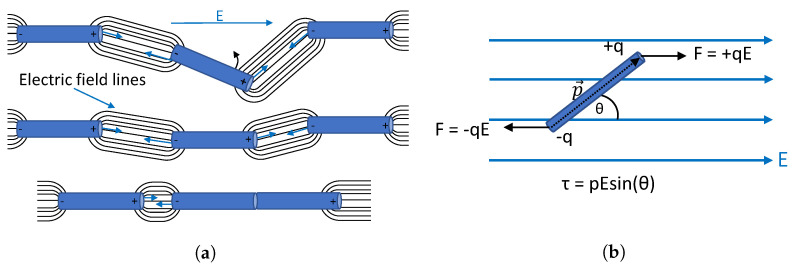
Carbon nanotube alignment principle. First, the nanotubes align themselves with the electric field due to the polarised nanotube ends. Secondly, the translational movement due to CNT-CNT attraction causes long connecting pathways to form between the electrodes. (**a**) CNT head-to-head alignment. (**b**) Principle of rotational forces on a dipole.

The alignment principle is based on the fact that the mere presence of dispersed nanotubes in a medium exposed by an electric field causes field inhomogeneities. The major force behind the CNT-CNT connections is due to the Coulombic attraction between oppositely charged ends of carbon nanotubes [25,26]. Due to the hexagonal interlinked carbon atom structure of the nanotubes, each carbon atom is bonded to its neighbours by three valence electrons, leaving one electron free to move [27]. The polarised CNTs have an opposite increased charge density at the outer ends, causing local electric fields that can cause movement of other nanotubes in close proximity (dielectrophoresis). This principle is shown in Figure 3a. This figure only presents the field lines connecting the CNTs. The field lines between the poles of the nanotube are removed for clarification. Due to the accumulated charges at the poles, these regions exhibit the highest field strength and are the “docking places” for other nanotubes. This effect thus yields the characteristic head-to-head CNT-connections, forming long nanotube pathways.

In the presence of an electric field, the conductive carbon nanotubes are polarised and experience a dipole moment. The induced torque acts on the carbon nanotube and aligns it with the electric field [28,29]. This process is graphically shown in Figure 3b. The maximum torque is present when the dipole is perpendicular to the applied electric field (maxτ). The smaller the angle between the polarisation vector and the electric field, the smaller the induced torque (minimum when sin(θ=0)=0). The alignment will continue until an equilibrium has been reached. This is either when the dipole is fully aligned or when the alignment force is not strong enough to facilitate the movement (weak electric field or too viscous a medium).

## 3. Analytical Model for the Piezoresistive Effect

Two main principles of piezoresistivity are expected to occur while the sensors are experiencing tensile strain:

- A loss of conductive networks due to the reorientation of CNTs within the polymer matrix. The CNTs change their orientation and position due to the applied strain. In general, it is assumed that the CNT-CNT connections start breaking up with increasing strain. As the polymer matrix is highly flexible, the effects are reversible with a decreasing strain. This relation is shown by Equation (1), where the applied stress (σ) causes the decrease in the interparticle distance *s*.
  (1) $$s=s_{0}\left(1-\epsilon \right)=s_{0}\left(1-\frac{\sigma }{E}\right)$$
- Electron tunnelling when the CNT-CNT distance is closer than 1.0 nm. The carbon nanotubes within this distance can conduct current, which with the increasing strain will abruptly stop when the distance becomes too great.

The more interesting effect due to the alignment of CNTs is the electron-tunnelling effect, which could theoretically increase the gauge factor by multiple orders of magnitude. The electric field causes the CNTs to rotate and align in the direction of the induced field. If the CNTs come close together (<1.0 nm), a current can flow between the CNT-isolation-CNT barrier. This tunnelling effect depends on the distance between the media and its barrier height. The tunnel resistance is shown in Equation (2), based on Simmon’s theory [30]:

(2) $$R_{tunnel}=\frac{V}{AJ}=\frac{h^{2}d}{Ae^{2}\sqrt{2m\lambda }}exp\left(\frac{4\pi d}{h}\sqrt{2m\lambda }\right)$$

**Figure 4:**
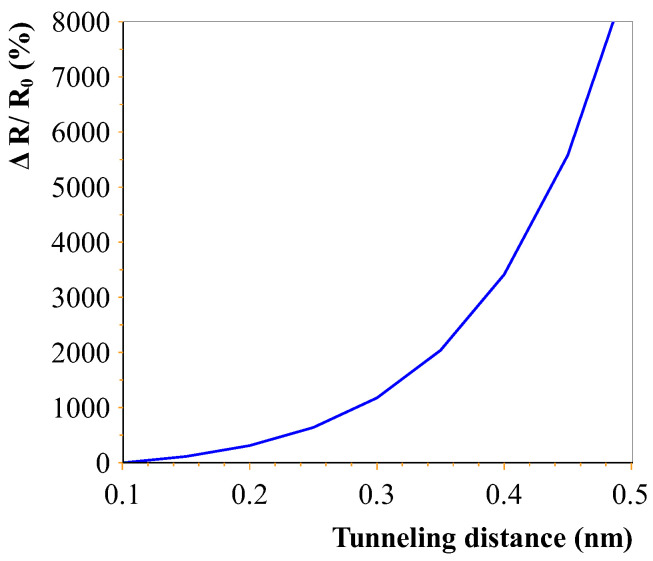
Relation between electron-tunnelling distance and the change in resistance for a single carbon nanotube pair. This relation follows from Equation (2). $R_{0}$ is the resistance value at a distance of 0.1 nm.

The cross-sectional area of the CNT is denoted by *A* (OD = 50 nm), tunnelling current density *J*, Plank’s constant *h*, tunnelling distance *d*, mass of the electron m and the barrier height λ. The barrier height, according to the literature, was approximated between 0.5 eV and 2.5 eV for epoxy [30]. The CNTs in the polymer experienced a poor stress transfer from the polymer matrix due to the large elastic mismatch (CNT: 270–950 GPa [31], PDMS: 0.57–3.7 MPa [32]) and weak interface strength [30]. Hence, the deformation of the CNTs due to the applied strain was assumed to be negligible, and the piezoresistivity was mainly attributed to the tunnelling effect. Figure 4 shows the relation between the tunnel distance and the corresponding change in resistance (based on Equation (2)). The exponential relation is clearly visible. High changes in conductivity occur when the distance between individual tunnelling CNTs is changed slowly. This effect is far greater than the regular piezoresistive property using conductive CNT networks that break up with an applied strain. Hence, it can be assumed that if high resistances are measured with large (nonlinear) resistance changes, this is attributed to electron tunnelling. This effect will outweigh the change in resistance due to disconnecting CNTs that change their orientation.

**Figure 5:**
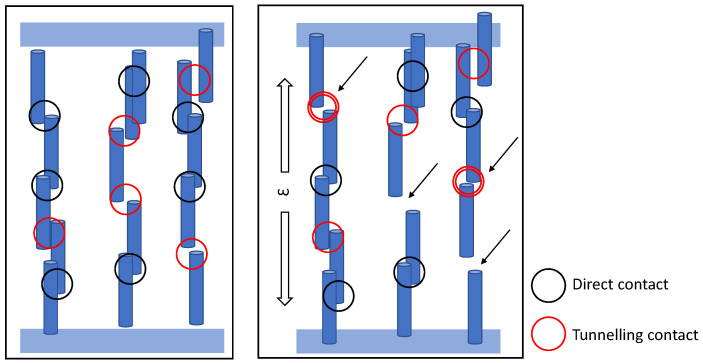
Analytical model principle. With an applied tensile strain, previously connected CNTs detach and become an electron-tunnelling connection, or previous electron-tunnelling connections become too large and result in a CNT-CNT disconnection. The arrows indicate changed connections.

A more detailed analytical model was created to show the effect of aligned carbon nanotubes and their CNT-CNT interactions via either direct contact or electron tunnelling. The large difference in sensitivity in the alignment direction and the orthogonal direction was validated by means of a complex analytical model. The general concept of the model is shown in Figure 5. The model consisted of two vertical electrodes with a specified number of carbon nanotubes (high-aspect-ratio rectangles). The nanotubes were placed vertically aligned in several conductive paths as shown in the figure. Variations in both *x* and *y* directions were included to ensure a slight random positioning. A valid electrical connection was determined when either the nanotubes were in direct contact or close enough for tunnelling (<2.5 nm in this model). The conductive paths were determined (which were classified as a valid connection) and the resistance was calculated for each connection. This was done either via determining the resistance of the connecting “wires” (R=ρ(L/A)) or by the exponential electron-tunnelling equation shown in Equation (2).

A summary and a brief overview of the analytical method is presented below.

- Parameter definition, including CNT dimensions, maximum tunnel distance and number or objects.
- Creation of rectangles (simplified CNTs). Two main electrodes at the top and bottom and a specified number of random or aligned rectangles.
- Determination of the closest distances between all rectangles. The rectangle borders are implemented for this, not the centre points.
- The shortest path is determined between the electrodes via Dijkstra’s shortest path algorithm.
- A strain on the sensors is simulated by changing the y-coordinates of all objects, just as with a real applied strain.
- The distances and shortest paths are determined once again for all applied strains.
- The resistance of each connection is determined, either via tunnelling or direct contact. Electron tunnelling is possible for distances lower than 2.5 nm, featuring the exponential relation between resistance and distance. The direct contact resistance is determined by the length and width of the connection.
- Finally, multiple parallel conductive connections are modelled and their parallel resistance is determined.

**Figure 6:**
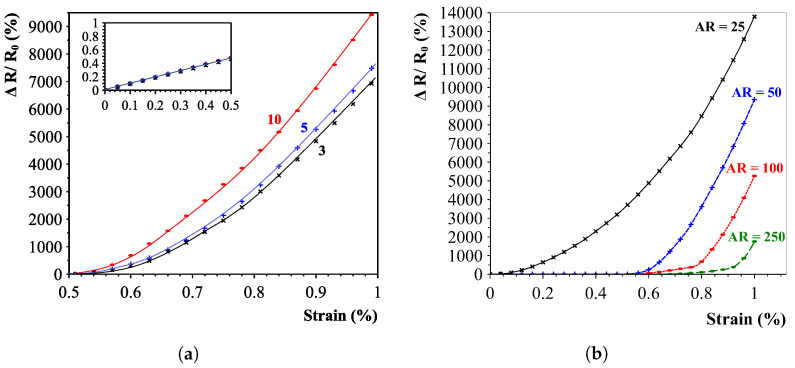
Analytical results to model the response of aligned carbon nanotube networks. (**a**) Influence on sensitivity with a varying number of CNT-CNT connections (10, 5 and 3). Simulations show an increase in sensitivity for higher number of connecting nanotubes (similar to wider interdigital electrode distances). (**b**) Influence on sensitivity by varying the aspect ratio of CNTs. A fixed electrode distance was chosen with varying-aspect-ratio CNTs. The initial linear low gauge factor response seems to lengthen with the increasing aspect ratio.The inset of (**a**) shows the linear initial low gauge factor response of up to 0.5%, after which electron tunnelling becomes more dominant, and the response drastically increases.

It is expected that after the alignment of carbon nanotubes in the polymer is complete, conductive paths have been formed. Initially, this would mean the nanotubes are in contact before the applied strain. The nanotubes are slowly pulled apart until the first connections break and electron tunnelling occurs and suddenly jumps the linear strain response to an exponential growth. This property is also seen in the analytical simulations and shown in Figure 6. The left figure shows the relation between the expected resistance change for different numbers of connecting CNTs. All results show an initial low gauge factor (≈1, see inset) up and until the strain is large enough and the direct connections break. The longer the CNT paths between the electrodes are, the higher the sensitivity appears to be. Figure 6b presents the effect of the aspect ratio on the sensitivity. This effectively confirms the previous figure because low-aspect-ratio connected CNTs feature more connections over the same distance and hence feature a higher sensitivity. Additionally, the aspect ratio correlates closely with the percolation threshold of the mixture. The relation can be mathematically represented by the relation shown in Equation (3) [33], which clearly shows the inverse dependency between aspect ratio and percolation threshold.

(3) $$\Phi_{pc}=\frac{\left(3+\delta_{d}^{2}\right)}{6AR}$$

**Figure 7:**
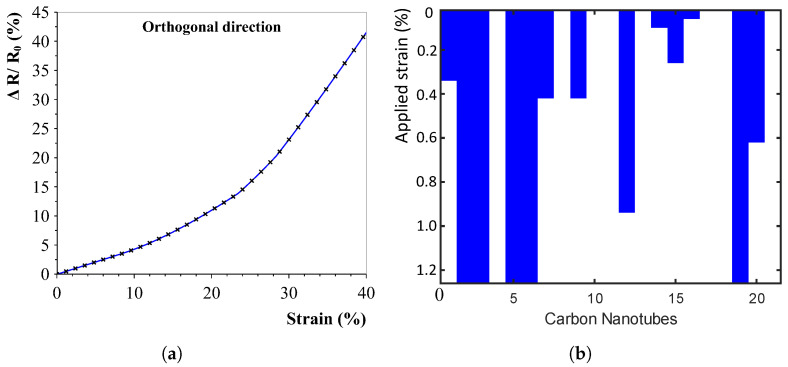
Numerical simulation results and model principle. (**a**) Strain sensitivity response by pulling orthogonal to the alignment direction. Responses show a far lower sensitivity than in the alignment direction shown in (**a**). (**b**) Visualisation of the available tunnelling connections (in blue) decreasing with the applied strain. Vertically, a decrease in tunnelling connections is visible due to the increasing strain. Left: low sensitivity in the orthogonal direction. Right: visualisation of the loss of connection (parallel to the alignment) with the applied strain. The figure shows the connections for a total of 20 carbon nanotubes on the *x*-axis and their present connection status (blue or white).

Ideally, if all vertically conductive paths are perfectly aligned, a strain in the orthogonal direction (horizontal in this case) should have no effect on the sensor. However, due to possible cross-connections, a small change is still expected. The results of the strain sensitivity in the direction orthogonal to the alignment direction is shown in Figure 7a, showing the low changes as suspected (please mind the larger applied strain of up to 40% on the *x*-axis).

The loss of connections is shown in Figure 7b. This map indicates all CNT-CNT electron-tunnelling connections (in this case 20). The connections, indicated by the blue colour, decrease with the increasing strain, causing the large increase in measured resistivity. After a certain amount of strain, all connections are too far away, and no conductance is measured.

## 4. Numerical Simulation for Nanotube Rotation

**Figure 8:**
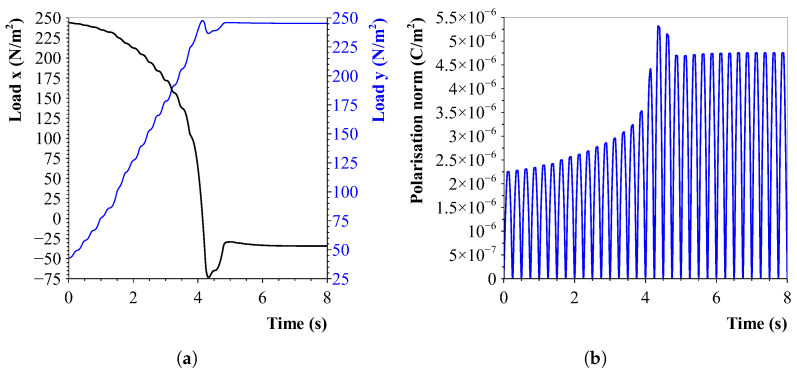
Applied forces on the carbon nanotube due to the applied electric field. (**a**) Load on CNT-ends while aligning in the electric field (in *y*-direction). Initially, the CNT is angled 30${}^{\circ }$ to the electric field. The load on the *y*-direction increases as the load on the *x*-direction decreases with the alignment. (**b**) Polarisation of the CNT during alignment in an electric field (2 kV/m AC, 2 Hz). The cyclic behaviour of the AC field is visible in the polarisation graph. An increase until the nanotube stabilises in the electric field is shown.

The concept of rotating CNTs inside a liquid medium using an external electric field was validated through a COMSOL Multiphysics model. The model consisted of a single CNT (with an outer diameter of 30 μm and a length of 600 μm) placed in a liquid (water) under the influence of a 2 kV/m, 2 Hz AC field. This first model featured these larger CNT dimensions in order to keep computation times short. Figure 8a illustrates the load on the nanotube in the *x*- and *y*-direction. The applied electric field is in the y-direction and the CNT has a starting angle of 30${}^{\circ }$ with respect to the *x*-axis. With alignment, the *x*-component of the load decreases, as the *y*-component increases. Eventually, the nanotube stabilises in the aligned direction. Furthermore, as depicted in Figure 8b, it can be observed that the nanotube becomes polarised, and the polarisation increases until an equilibrium has been reached.

**Figure 9:**
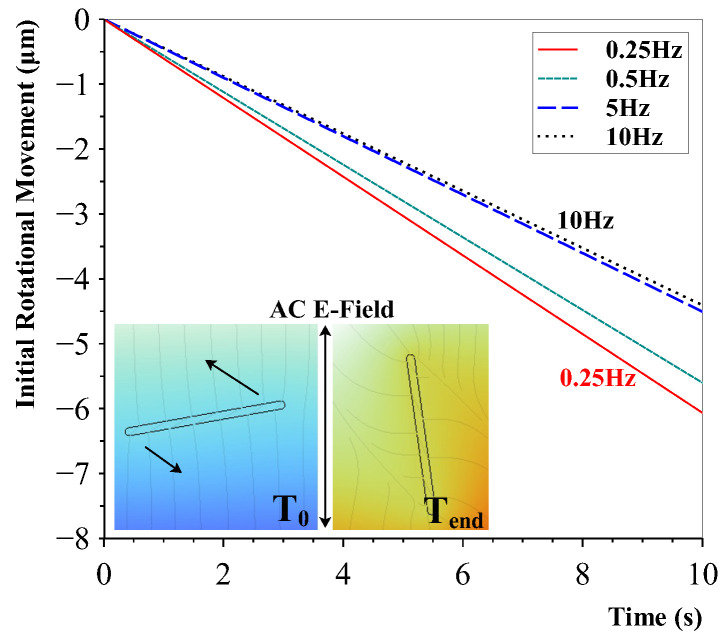
Rotational movement of CNTs in a polymer with different electric field frequencies. The higher frequencies show a lower rotation movement. The inset shows the rotation of the carbon nanotube. The colour differences in the background are due to the AC field.

In a more detailed and realistic model, the same CNT dimensions as the real CNTs used in this research were considered: an outer diameter of 50 nm and a length of 5 μm. The influence of frequency with a fixed voltage was investigated, and the results are presented in Figure 9. The results indicate that the rotational movement of the CNT decreases with the increasing frequency.

The rotational movement of the CNT is not a straight line and corresponds to the applied frequency waveform. For clarity, a straight line is shown to visualise the differences. The initial movements were linear until the rotation exceeded a certain threshold, after which the rotation decreased.

It is believed that the CNTs have a certain threshold after which they have enough energy to rotate within the liquid. The highest polarisation occurs at the peaks of the AC voltage, during which the length surpassing this threshold decreases with the increasing frequency. Therefore, it is thought that a frequency too high may not be able to fully polarise the CNT in time and stop or slow down the alignment.

**Figure 10:**
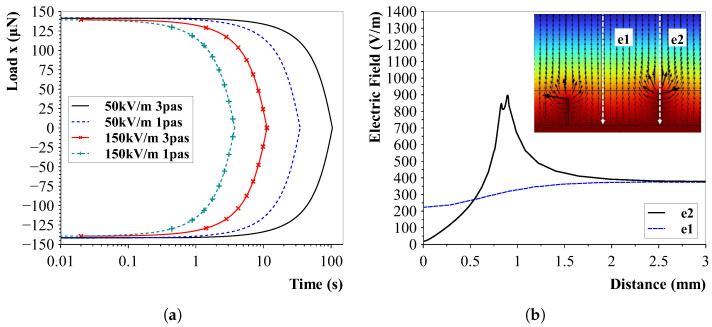
Numerical simulation results of rotating carbon nanotubes in an electric field. (**a**) Influence of viscosity and field strength on the alignment of CNTs. Viscosities were chosen to correspond to uncured PDMS. (**b**) Effect of connected CNTs to the outer electrodes. The nanotubes cause locally high electric fields that disturb the alignment.

The influence of the viscosity and electric field strength on the CNT alignment is demonstrated in Figure 10a. The figure illustrates the load on both ends of the CNT. Since the opposing ends rotate in opposite directions, the relation between the loads have opposite signs. The crossing point indicates the parallel position of the CNT in relation to the applied field.

In the simulation, the viscosities and parameters were selected to correspond to the properties of PDMS. The data revealed that an increase in viscosity (from 1 to 3 pas) resulted in a longer alignment time. Conversely, an increase in electric field strength decreased the alignment time. These observations suggest that a higher viscosity requires more time for the CNTs to align with the applied field, while stronger electric fields expedite the alignment process.

This information provides insights into how viscosity and electric field strength can influence the alignment kinetics of CNTs in a liquid medium. Additionally, the first values of the needed field strengths and frequencies for the experiments are now known.

If carbon nanotubes attach to the conductive electrodes, they can create local areas of increased electric field strengths and cause alignment imperfections. This effect is simulated and demonstrated in Figure 10b, where the increase in field strength (e2) is clearly visible. It can also be clearly seen that the direction of the electric field strength changes. This is due to the fact that the growth of CNT connections changes over time, leading to variations in local electric field strengths and directions.

## 5. Experimental Results

**Figure 11:**
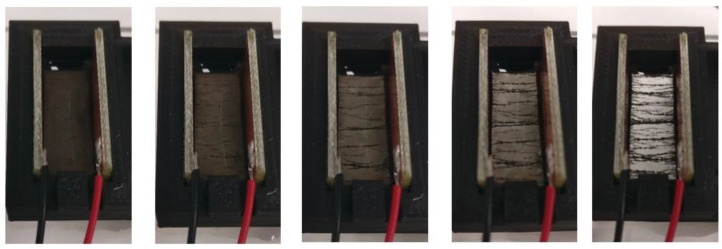
Carbon nanotube alignment in DI water as a proof of concept. The electrodes are placed directly into the solution.

Carbon nanotubes were mixed in deionised water and aligned with two electrodes on either side in a 3D-printed mould (1 cm spacing). The results are shown in Figure 11. It can be seen that the first homogeneous solution is nontransparent, and after the alignment continues, more and more thick connections form. At the end, it looks like all CNTs are in thickly formed “highways” between both electrodes. This test was performed by placing the electrodes directly into the solution, enabling for a current to flow once connections were formed. The applied AC voltage was 13.3 kV/m at 1 Hz.

**Figure 12:**
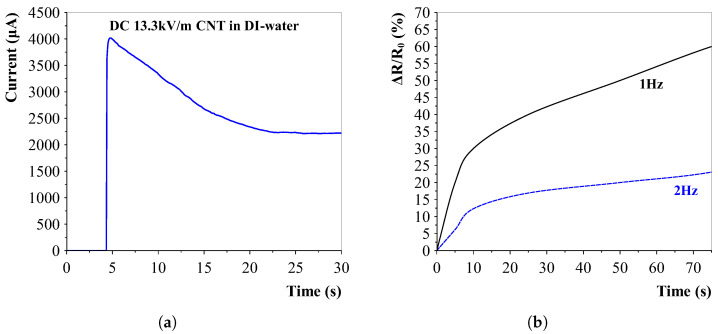
DC and AC Alignment in deionised water. (**a**) DC alignment shows a migration of carbon nanotubes. The conductivity decreases due to the nanotube migration. (**b**) Frequency influence on CNT alignment for 0.02 wt% in deionised water. In PDMS, a lower electric field frequency shows a faster alignment.

Using a DC voltage yielded the same alignment effect but with the major disadvantage of slowly pulling all charged CNTs to a single side. This was experimentally proven by applying a DC voltage of 13.3 kV/m and measuring the current during alignment. Figure 12a clearly shows a very sharp increase in current, after which the CNTs start to pull to one side and the current decreases slowly until it stabilises. It is thought that at this saturation point, there is a formed connection present between both electrodes via the sidewalls of the mould.

It is expected that the migration of nanotubes when positioned in a DC electric field is caused by the initial charge of the nanotubes. Depending on the manufacturing process of the carbon nanotubes, they can become charged. For example, if the CNTs are treated with acidic functional groups, they can become positively charged. Basic groups can cause negatively charged CNTs [34]. This has to do with the fact that acidic groups tend to donate protons and become negatively charged. The donated protons adhere to the CNT surface, giving them a net positive charge, which can cause the translational movement of carbon nanotubes. This effect is the main reason why low alternating voltages were implemented in this research to align the carbon nanotubes.

The CNT concentration was lowered even more to try and visualise the effect of the alignment on the conductivity of the solution. Two different frequencies were applied and the results are depicted in Figure 12b. Results confirm previous experiments that the alignment with higher frequencies (1 vs. 2 Hz) decreases. The most controllable alignment speed seems to be with the lower frequencies, which corresponds to the performed numerical simulations.

### 5.1. Characterisation of Alignment Parameters

After the initial experiments, the alignment parameter characterisation was performed with a manufactured mould featuring an electrode distance of 3 mm and a length of 1.5 cm. Two alignment methods were characterised: with the direct current application (electrodes inside the liquid) or an indirect alignment where the electrodes were isolated from the liquid. The alignment process was investigated for the following parameters: electric field strength, alignment time, indirect vs. direct alignment and frequency influence.

For the piezoresistive layer, PDMS with a 1:5 curing agent ratio was chosen. Normally, the standard ratio for the curing agent is 1:10. However, in this case, the proportion of the curing agent was increased to the maximum value that still yielded proper layers, which was a ratio of one to five. This adjustment was made to create the least viscous solution possible, which is beneficial for the alignment process.

#### 5.1.1. Electric Field Strength

The necessary electric field strength required to induce optical alignment of CNTs was investigated. A fixed frequency of 1 Hz was used for this study. If, after 60 min, no optical change in CNT alignment was observed, it was assumed that the applied field strength was not sufficient. Results showed a very precise window of field strength needed for aligning the CNTs in PDMS. An induced electric field of 460 kV/m did not result in an optical change in the CNT orientation, while with 500 kV/m, alignment occurred almost instantly.

As suspected, the speed of alignment increased with higher field strengths. To achieve better control in the alignment process, a field strength of 480 kV/m was chosen. This specific field strength produced an optical change in the CNT alignment after several minutes, which was deemed feasible for later wafer fabrication and sensor production.

#### 5.1.2. Alignment Time

**Figure 13:**
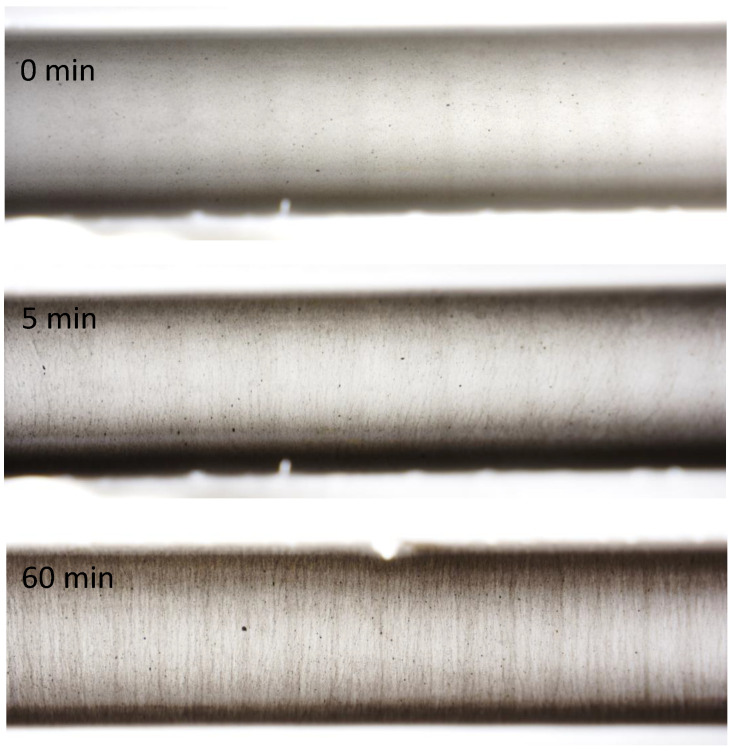
Carbon nanotube alignment for different durations (0, 5 and 60 min), with an electrode distance of 3 mm. First, a randomly dispersed solution is visible. After 5 min, the first conductive paths are visible, which become more pronounced after 60 min.

The CNT-polymer samples were subjected to an indirect electric field of 480 kV/m at 1 Hz for up to 60 min. Microscopic images were taken at intervals of 5 min, capturing the starting position, alignment after 5 min and alignment after 60 min, as shown in Figure 13. The results clearly demonstrate a noticeable change in alignment even after just 5 min, with a more pronounced effect after 60 min. As the electric field is applied for longer durations, the CNTs agglomerate and create thicker carbon nanotube bundles.

Furthermore, it was found that as the CNT weight percentage (wt%) in the polymer increased, the alignment of CNTs took longer. Beyond a certain CNT concentration, no more visible optical alignment was observed due the large clusters in the mixture.

#### 5.1.3. Indirect vs. Direct Alignment

**Figure 14:**
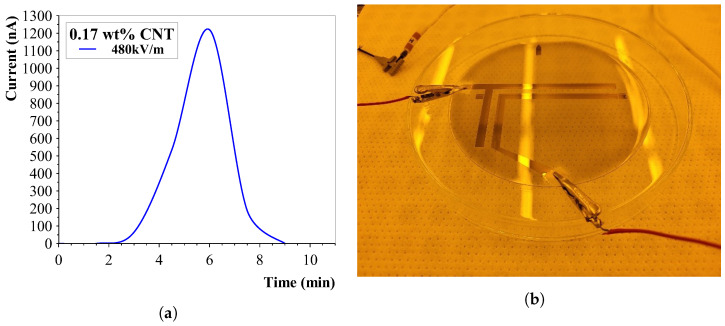
Carbon nanotube alignment results and alignment setup. (**a**) Measured current during the direct-method alignment process. The high voltages and the created connection between the electrodes caused a electrical short circuit, destroying the nanotube alignment. (**b**) Wafer alignment setup. The large parallel gold electrodes are the alignment electrodes. Between them, the nanotubes are aligned. The polymer was manually removed in order to have an electrical contact between the electrodes and the clamps connected to the high-voltage generator.

As mentioned before, two alignment methods are possible. Either the electrodes are placed in the solution, enabling a current to flow between the electrodes once conductive paths are formed, or electrodes are isolated. Both methods were investigated, and the results were clear. The indirect method was able to align the CNTs in the same (optical) way as the direct method. However, difficulties arose with the direct method, specifically with the sudden connection between both electrodes and the high required voltages. Figure 14a shows a measurement performed while aligning the CNTs in PDMS. After some time, there was an increase in current flow, but it suddenly dropped back to zero. At the instant a connection was created between both poles, the released energy due to the short circuit was so significant that a small visible shock wave ran through the solution, instantly curing the PDMS at the location of the short circuit and destroying the alignment.

Additionally, with the direct alignment method, it was observed that after the solution was optically aligned, the edges on either side started moving turbulently. A circular motion was visible on either side, while no movement was visible in the middle. It is theorised that this effect is caused by the change in the local electric field orientation due to the CNTs attached to the electrodes, as previously discussed and shown in Figure 10b.

Due to these results and the challenges encountered with the direct method, the indirect method was chosen for aligning the CNTs in the wafer-fabricated sensors. The indirect method demonstrated a successful carbon nanotube alignment without the associated complications.

#### 5.1.4. Frequency Influence

Frequencies of 1 Hz, 10 Hz, 100 Hz and 1 kHz were investigated with a fixed voltage of 480 kV/m for up to 60 min. The time it took to optically move and align the CNTs was taken as a comparative study between the frequencies. Results are shown in Table 1. Similar to the simulation results, the alignment time increased with the increasing frequency.

### 5.2. Sensor Results

Sensors were fabricated with four different CNT concentrations (0.04, 0.08, 0.17 and 0.33 wt%) to investigate their influence on sensor quality. Typically, concentrations below 0.2 wt% were considered to be nonconductive. Therefore, any measured conductivity change was attributed to the alignment process.

The glass wafers (featuring the isolated gold alignment electrodes) were placed on the spin-coater and a 65 μm thick CNT-PDMS layer was deposited. The wafer was placed on an upside-down Petri dish where both electrodes were connected to the Labsmith HV3000 high-voltage generator, which was set to 480 kV/m at 1 Hz. The alignment time was set to 60 min, after which the electric field was turned off, and the PDMS layer was slowly cured at 90 ${}^{\circ }$C for 45 min. The alignment setup is shown in Figure 14b.

**Figure 15:**
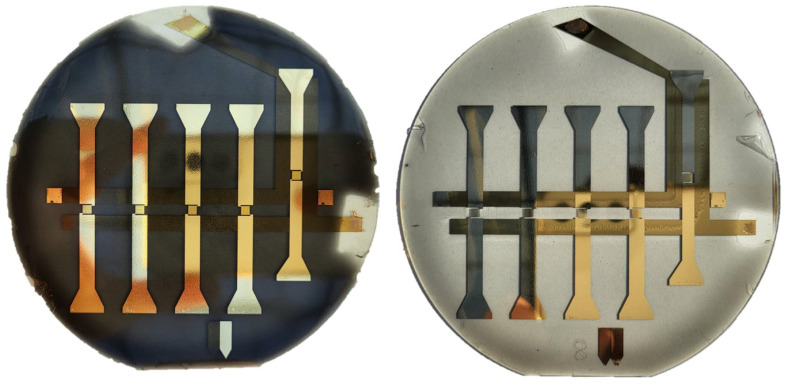
Strain sensors on the wafer. Different CNT concentrations cause changes in transparency (left 0.33 wt%, right 0.08 wt%).

Depending on the added CNT concentration, the layers were optically transparent (<0.2 wt%), or almost black-coloured (0.33 wt%), as shown in Figure 15. On the right side of the figure, the bottom horizontal alignment electrodes are still visible. On top, the vertical strain sensor with its large connections is shown.

**Figure 16:**
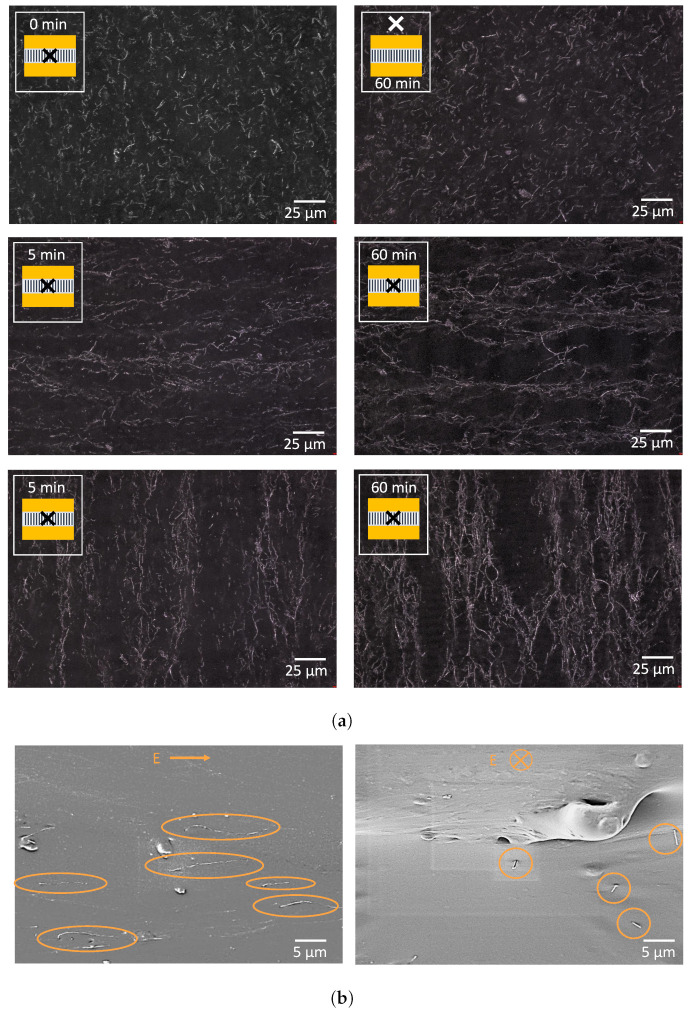
Microscopic results depicting the carbon nanotube alignment in the cured PDMS layer. (**a**) Optical results of CNT alignment in PDMS. The insets indicate the location of the picture (outside alignment area, on the electrodes or in the alignment area). More agglomerated conductive networks form after 60 min. A clear alignment orientation is detectable. (**b**) Scanning electron microscope view of aligned CNTs, viewed from both directions. The left picture shows a parallel view to the alignment direction, as the right image shows a frontal view of the aligned nanotubes. The direction of the electric field is indicated in the top centre part of the figures.

The alignment was inspected via an optical-light microscope. The results immediately before and after alignment are shown in Figure 16a. The carbon nanotubes are randomly oriented before the alignment. After 5 min, a clear first alignment of the CNTs is detected, with stronger pathways after 60 min. Multiple sensors were manufactured on a single wafer. Half of the sensors were aligned in the horizontal direction, the other half in the vertical direction, as shown in Figure 16a. This figure also shows that even after 60 alignment minutes, no movement was visible outside the alignment area (top right image). With the help of the scanning electron microscope, the alignment of the CNTs was confirmed inside the material by looking with a parallel view, or directly from the front, as shown in Figure 16b. Only a few carbon nanotubes are visible due to the very low concentrations of carbon nanotubes.

**Figure 17:**
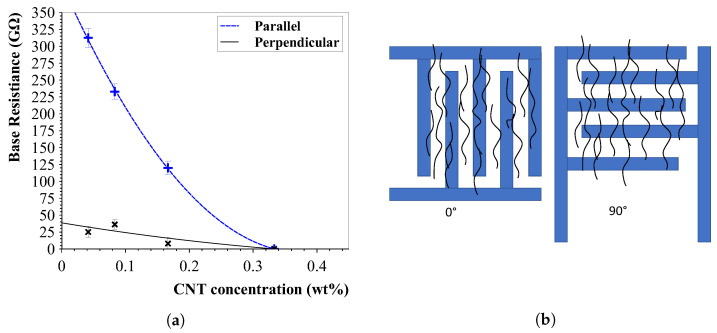
Base resistances of sensors with different nanotube concentrations (left) and view of the interdigital electrode setup (right). (**a**) Initial resistance of strain sensors. The perpendicular IDE structures yield more connections, as suspected, and show greatly decreased resistances as the parallel counterpart. A higher CNT wt% results in a lower sensor resistance, as expected. (**b**) Sensor design with two IDE orientations. The perpendicularly rotated IDE structure to the alignment direction causes more CNT pathway connections and is expected to be more sensitive to strain than the parallel setup.

The unidirectional behaviour of the sensor was investigated by creating two different IDE-design structures on top of the sensing layer. The sensor is based on the fact that with applying strain, the distance between the conductive particles increases and the conductivity decreases. Ideally, with the aligned CNTs, the sensitivity is increased in the preferred direction and remains unchanged in all others. This is explained more clearly in Figure 17b. The carbon nanotubes in the left part are aligned in the same direction as the interdigital electrodes. If a strain is applied in the vertical direction, the CNTs are pulled apart, but the conductivity difference of both parts of the IDE is not greatly affected. If the IDEs are rotated 90${}^{\circ }$ and the aligned CNTs cross both IDEs and connect them together, the resistance change should be increased.

#### 5.2.1. Base Resistance Change

The base resistance of all samples was measured and is plotted in Figure 17a. The sensors with the parallel IDE orientation featured a far greater base resistance than its counterpart. This is expected as it is thought the parallel IDE orientation has less connections between them via the aligned CNTs. With an increasing wt% in the piezoresistive layer, a decrease in base resistance was measured. Without any carbon nanotube alignment, the solution showed a resistance of >1 GΩ.

#### 5.2.2. Two-Phase Piezoresistive Response

The carbon nanotube-aligned piezoresistive strain sensors exhibited, similar to the analytical simulations, a two-phase response. For the first small strain range, the electron-tunnelling effect appeared not to be the dominant factor and was combined with the regular piezoresistive effect. It is theorised that a large part of the CNT-CNT connections stay connected and only shift, resulting in linear resistance changes. After a certain threshold was reached, electron tunnelling occurred due to the increased change in disconnecting CNT-CNTs, drastically increasing the response of the sensors up until a point where no more conductive paths were present.

**Figure 18:**
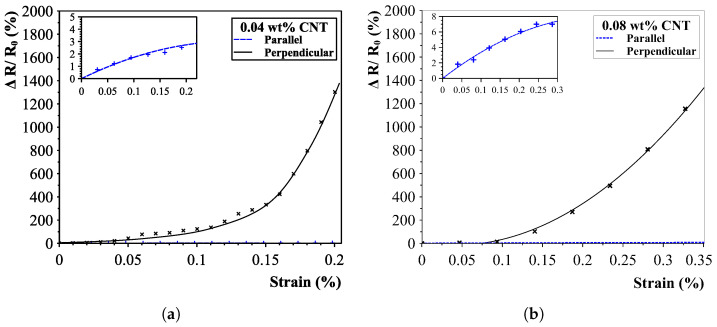
Strain results for different carbon nanotube concentrations with an interdigital electrode distance of 200 μm. A clear distinction in sensitivity is present between the parallel and perpendicular IDE orientation. (**a**) Results for 0.04 CNT wt% sensors. (**b**) Results for 0.08 CNT wt% sensors.

**Figure 19:**
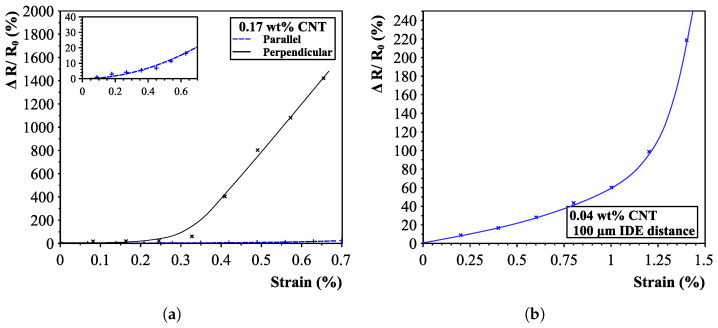
0.17% sensor result with a 200 μm interdigital electrode distance (**a**) and result for a 0.04% 100 μm distance (**b**).

Results of all sensors clearly indicated a great sensitivity change within the measured strain regions (up to 0.7%). Results with an interdigital electrode distance of 200 μm are shown in Figure 18 and Figure 19a. Large differences in sensor responses are present for both measured directions. The unidirectional sensitivity of the sensors is proven when looking at the responses for both the parallel and perpendicular directions. Small sensor responses in the parallel direction are caused by the cross-connections in the aligned pathways.

Additionally, the effect of the interdigital electrode spacing is shown in Figure 19b, confirming analytical calculations that shorter distances yield less sensitive sensors. The maximum found k-factor for the linear region was 60 (in contrast to 550) and saturating at almost a 300% resistance change (instead of 1600% for the 200 μm interdigital electrode spacing sensors). However, results do show a smoother response and far greater sensitive region than the 200 μm IDE-spaced sensors (1% to 0.25% strain).

**Figure 20:**
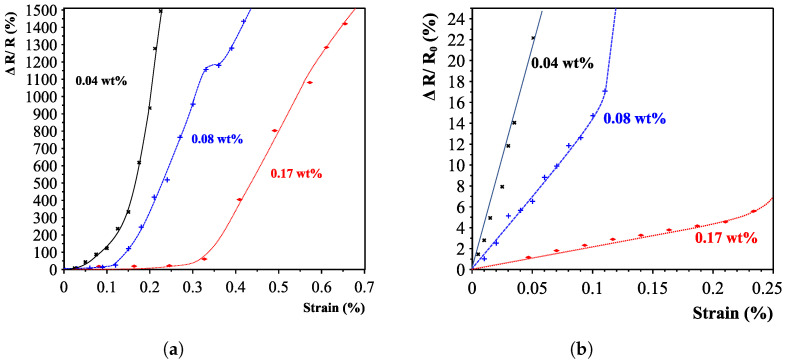
Sensor responses for different carbon nanotube concentrations with a zoomed-in version of the initial sensor response for the first strain region of 0–0.25%. (**a**) Complete sensor response overview for different nanotube concentrations. (**b**) Zoomed-in of the initial first-phase piezoresistive responses.

It was found that the concentration of carbon nanotubes does not play a major role in the maximum achievable resistance change. All nanotube concentration sensors saturated around a resistance change of 1600%. This is mainly caused by the fact that all aligned sensors featured a starting resistance on the same order of magnitude, and the upper limit was set by the maximum measurable resistance of the high-precision multimeter (<1 TΩ). Results did show, however, that higher concentrations yielded longer linear regions, as can be seen for the 0.17 wt% strain (up to 0.3% strain, see Figure 20). It is thought that with higher concentrations, thicker and more bundled networks are created, needing more applied tensile strain to reach the second “electron-tunnelling” phase.

Results in this study give very high gauge factors, which correspond to the theoretical and analytical findings of creating a piezoresistive polymer based on tunnelling electrons that hop between individual carbon nanotubes. The large initial resistances of the sensors is attributed to the fact that due to the alignment of CNTs and the very low percentages of added nanotubes, only a few very thin (<10 μm) and long (200 μm) connections are created. This, in combination with possible CNT-CNT connections via electron tunnelling, gives rise to the high resistances.

#### 5.2.3. Piezoresistive Tunnelling Effects

**Figure 21:**
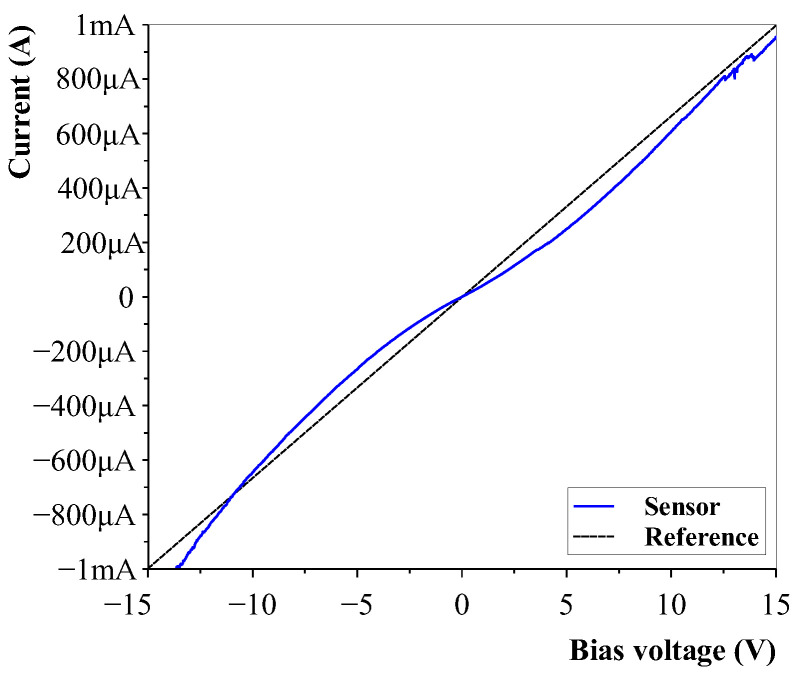
Nonlinear sensor behaviour indicating the presence of electron-tunnelling connections in the carbon nanotube–polymer sensor layer.

Both the numerical results and the sensor results showed an exponential relation between the applied strain and resistance change and showed that very high gauge factors were possible due to electron tunnelling. A bias voltage sweep was applied to the sensors to confirm the sensors did indeed experience electron tunnelling. A 15 kΩ resistor was taken as a reference. The results of the nonlinearity of the sensors, indicating a nonlinear resistance due to tunnelling effects, is shown in Figure 21.

**Figure 22:**
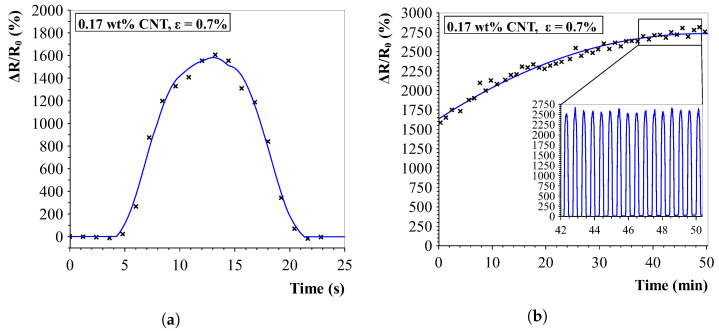
Aligned sensor response to (repeated) strain exposure. (**a**) Individual strain cycle. The sensors show similar responses for the tensile strain phase and relaxing of the sensor. (**b**) Cyclic strain response. An initial small drift upwards is visible, after which the sensor stabilises and shows a repeatable response.

The dynamic stability and repeatability of the strain sensors was investigated by exposing the sensors to a repeated strain of 0.7% for up to 100 cycles (Figure 22). Responses showed a slight upward drift until the sensor appeared to stabilise and showed a highly stable dynamic response.

## 6. Discussion

**Figure 23:**
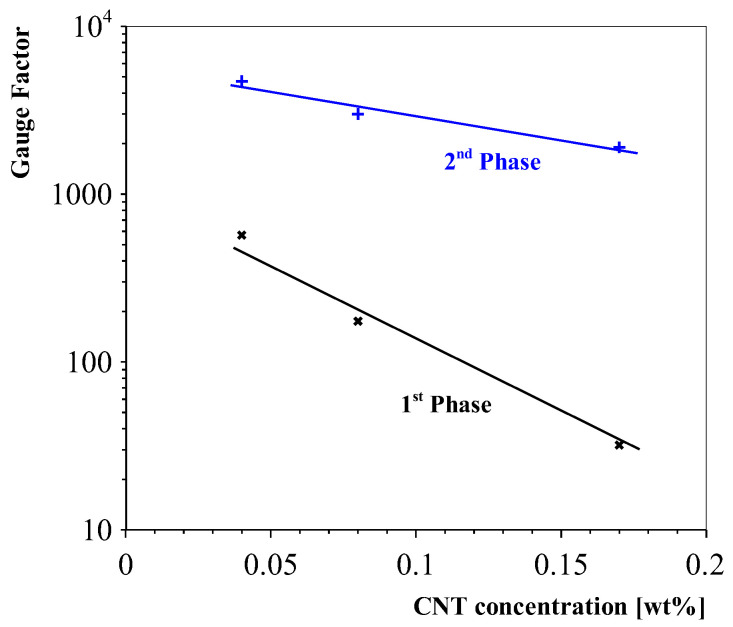
Determined gauge factors divided into the two piezoresistive phases for different carbon-nanotube weight percentages (0.04–0.17).

This research presented carbon nanotube-based strain sensors that featured a preferential direction due to the aligned conductive pathways. The unidirectional property was confirmed by characterising the sensors with two oriented interdigital electrodes on top of the sensing layers, as well as optical and scanning electron microscope images. The interdigital structures that were perpendicularly aligned to the alignment direction featured gauge factors of up to k ≈ 4500 (depending on the nanotube concentration). The interdigital electrodes featuring a parallel orientation to the alignment direction were found to have a gauge factor of around ≈22. The sensors featured a two-phase piezoresistive response. Initially, the sensors showed a linear response region, after which the electron-tunnelling effect became more dominant, resulting in a high gauge factor response. This is summarised in Figure 23. The sensors show a decrease in sensitivity with the increasing carbon nanotube concentration. This effect is similar to that with randomly aligned carbon nanotube sensors [5,10,35]. The reasoning behind this effect is that increasing the nanotube contents leads to more agglomerations and lowers the sensitivity [10].

The base resistance of the aligned sensors also showed a clear distinction in conductivity between the parallel and perpendicular IDE structures, indicating a change in the CNT-orientation network.

The behaviour of the rotating carbon nanotubes and the expected piezoresistive effect was analytically and numerically modelled and confirmed later by experimental results. The analytical model showed high gauge factors reaching up to 4000 for aspect ratios of 100–250, corresponding to the experimental values. The analytical model showed an inverse relation between the number of nanotube connections and sensitivity, which was proven to be identical for the fabricated sensors in this research. The numerical simulation showed the order of magnitude necessary for the rotation of carbon nanotubes due to the presence of an electric field. The reasoning for the effect of the turbulent alignment with the direct current alignment method was also shown in the numerical model and experimental results.

The alignment of carbon nanotubes is greatly influenced by the field strength, frequency and time. An alternating field stops the nanotubes from migrating toward an electrode, and a longer time creates thicker agglomerated nanotube bundles. However, due to the cross-connections between the conductive pathways, an absolutely unidirectional sensitive sensor is not possible yet. Future research will investigate the possibility to align functionalised carbon nanotubes to enhance the alignment and improve the unidirectional sensor properties. The functional groups in the nanotubes cause a repulsive interaction between the aligning CNTs, increasing the alignment quality and minimising the cross-sensitivity. Another advantage is the fact that the functional groups increase the charge in the carbon nanotubes, increasing their dipole moment [36].

Additionally, first results showed an increase in the sensors’ sensitive region by decreasing the interdigital electrode distances. Future research could utilise this property to create CNT-aligned sensors with far greater (linear) sensitive regions.

## Figures and Tables

**Table 1:** Time to optical alignment of CNTs in PDMS polymer.

Frequency	Time to Align (min)
1 Hz	3
10 Hz	18
100 Hz	>60
1000 Hz	>60

## Data Availability

Not applicable.
